# Fates of nutrient elements and heavy metals during thermal conversion of cattle slurry-derived anaerobic digestates

**DOI:** 10.1186/s40643-024-00828-7

**Published:** 2024-12-30

**Authors:** Daniel J. Lane, Olli Sippula, Jorma Jokiniemi, Mikko Heimonen, Niko M. Kinnunen, Perttu Virkajärvi, Narasinha Shurpali

**Affiliations:** 1 Commonwealth Scientific and Industrial Research Organization (CSIRO), Queensland Centre for Advanced Technologies (QCAT), 1 Technology Court, PO Box 883, Pullenvale, Kenmore, QLD 4069 Australia; 2 Department of Chemistry, University of Eastern Finland, Joensuu, FI-80130 Finland; 3 Department of Environmental and Biological Sciences, University of Eastern Finland, P.O. Box 1627, Kuopio, FI-70211 Finland; 4 Production Systems Unit, Grasslands and Sustainable Agriculture Group, Natural Resources Institute Finland (Luke), Maaninka, FI-71750 Finland

**Keywords:** Ash utilization, Combustion, Gasification, Pyrolysis, Phosphorous recovery, Volatilization, Biochar

## Abstract

Thermal processes are emerging as promising solutions to recovering phosphorus and other nutrient elements from anaerobic digestates. The feasibility of nutrient element recovery depends largely on the fates of nutrient elements and heavy metals during thermal processing. This study assesses the partitioning of macronutrients (N, P, K, Na, Ca and Mg) and heavy metals (Zn, Cu, and Mn) between condensed and gaseous phases during thermal conversion of cattle slurry digestates in gas atmospheres of pyrolysis, combustion, and gasification processes. This study also assesses the chemical forms of macronutrients retained in combustion ashes. The partitioning of elements between condensed and gaseous phases was quantified by mass balances based on elemental analyses of char and ash residues. The char and ash residues were prepared in a fixed-bed, batch reactor at temperatures within the range 800–1000 °C. Powder X-ray diffraction was used to identify the chemical forms of macronutrient elements in combustion ashes. Volatilisation of P was low (< 20%) when the digestates were heated in inert and oxidising atmospheres, whereas a reducing atmosphere volatilized P to a major extent (~ 60% at 1000 °C). Oxidising atmospheres increased volatilisation of N but suppressed volatilisation of K, Na, and Zn. Volatilisation of the following elements was low (< 30%) in all investigated operating conditions: Ca, Mg, Mn, and Cu. The combustion ashes contained both high concentrations of P (around 7 w/w%) and acceptable concentrations of regulated heavy metals (Cu, and Zn) for application on agricultural and forest soils in Finland. Phosphorous was retained in the combustion ashes in the form of whitlockite. This form of P is expected to be available to plants when the ashes are added to soil.

**Supplementary Information:**

The online version contains supplementary material available at 10.1186/s40643-024-00828-7.

## Introduction

The most commonly used process to convert biomass wastes into biogas, anaerobic digestion, generates large quantities of a by-product called “anaerobic digestate”. This material is produced in the form of a slurry, which predominately consists of water, undigested organic material, and inorganic minerals. Until now it has been a common practice to apply anaerobic digestates to soils since it is often rich in phosphorous, nitrogen and other essential plant macronutrients (Lukehurst et al. 2010; Möller and Müller 2012). However, there is increasing concern over the environmental impacts of this practice.

Anaerobic digestates that have been derived from animal excrements generally contain a few percent N (Sørensen et al. 2011). Application of anaerobic digestates to soils can cause nutrient leaching into nearby water bodies and high emissions of nitrogenous pollutants, particularly nitrous oxide and ammonia, into the atmosphere (Nkoa 2014). Nitrous oxide is a strong greenhouse gas that causes depletion of ozone in the atmosphere (IPCC, 2022). Emissions of ammonia have adverse impacts on air quality and can contribute to eutrophication of surface waters (Nkoa 2014). Since anaerobic digestates tend to contain high concentrations of P and N in chemical forms that are readily leached (Güngör and Karthikeyan 2005; Svoboda et al. 2013), rapid nutrient run-off from fields that have been treated with anaerobic digestates have been reported (Nkoa 2014). Anaerobic digestates can also contain various pathogens, e.g., *Salmonella spp.*, and pharmaceuticals which threaten the health of crops, livestock, and humans (Bagge et al. 2005; Li et al. 2018). Direct application of anaerobic digestates to soils has potential to cause significant damage to the environment, particularly in areas where regional animal production has led to a considerable surplus of manure or where anaerobic digestates are produced in large quantities (Potter et al. 2010). This is motivating research into more sustainable utilisation options for anaerobic digestates.

Anaerobic digestates can potentially be used as feedstocks for thermal processes that produce one or more of the following products: fertilisers, heat, power, fuels, and chemicals. Technologies based on thermal processes, particularly combustion, pyrolysis, and gasification, have long been used to extract value from biomass and are well-established with large-scale installations in operation worldwide. Thermal processes can remove nitrogen from, and destroy harmful pathogens and pharmaceuticals contained within, anaerobic digestates (Leppälahti and Koljonen 1995; Seeker 1990; Winter et al. 1999). Furthermore, thermal processes result in a substantial reduction in total solids volume. Valuable inorganic nutrient elements that already exist in anaerobic digestates in high concentrations, e.g., P and K, have potential to become substantially enriched and better stabilised in the ash (or char) residues following thermal processing. The potential to utilise these residues for production of mineral fertilisers and biochar soil ameliorants has been investigated by previous researchers (e.g. Thygesen and Johnsen 2012; Catenacci et al. 2022; Basinas et al. 2023). Despite this, the application of anaerobic digestate-derived chars and ashes to soils is seldom practiced at large scale.

Recent studies have compared the potential of different thermal processes, including combustion, gasification, and pyrolysis, for nutrient recovery from sewage sludges (e.g., Thomsen et al. 2017; Zhu et al. 2022; Hannl et al. 2024). These studies have shown that the type of thermal process and operating conditions impacts the recovery, chemical speciation, and bioavailability of the nutrients. On the other hand, limited work has been done to compare the potential of thermal processes for nutrient recovery from cattle slurry digestates.

Detailed knowledge of the fates of valuable nutrient elements under different thermal processing conditions of anaerobic digestates is needed for the development of effective thermal recovery processes. In addition, there is a need to study the fates of heavy metal contaminants, particularly Zn, Cu, and Mn, during the thermal processing of cattle slurry digestates, since these elements can prohibit the use of ash (or char) residues as a fertiliser or soil amendment if they become too concentrated in the target ash (or char) fraction. In general, solid ash and char residues vary greatly in their physical and chemical properties depending on where and how they are collected in thermal processes (Dahl et al. 2009; Lind et al. 1999). The partitioning of nutrient elements and heavy metals into different ash streams, e.g., bottom ash, fly ash, and air pollution control residues, depends largely on the extent to which they volatilise from the biomass bed (Clarke and Sloss 1992; Nzihou and Stanmore 2013; Obernberger et al. 1997; Kortelainen et al. 2015). This in turn depends on the chemical composition of the biomass feedstock and on operating parameters, especially temperature and gas atmosphere (Lane et al. 2015a, b; van Lith et al. 2008).

Current knowledge of the volatile behaviour of nutrient elements and heavy metals during thermal processing of biomass is largely rooted in studies based on conversion of feedstocks which typically have low concentrations of P, e.g., coal and wood (e.g., Bläsing and Müller 2010; Lind et al. 1999; van Lith et al. 2008). Several recent studies (e.g., Hedayati et al. 2021; Ren and Li 2015; Skoglund et al. 2014) have shown that the ash-forming constituents of these feedstocks follow different transformation pathways to the ash-forming constituents of biomass feedstocks that contain high concentrations of P. For example, the alkali metals (K and Na), which typically form sulphates, chlorides, silicates, and carbonates during combustion of low-P feedstocks, preferentially form phosphates during the combustion of P-rich feedstocks (Grimm et al. 2012). Reported differences in ash behaviour such as this, have been attributed to the high affinity of P for base metal cations (Boström et al. 2012) and can be expected to significantly impact on the partitioning of nutrient elements and heavy metals within thermal processes.

Despite recent advancements in scientific understanding on ash formation with P-rich fuels (Hedayati et al. 2021; Falk et al. 2020; Nordin et al. 2020; Häggström et al. 2023; Lidman et al., 2023) it is still difficult to accurately predict the extent of volatilisation of macronutrient elements and heavy metals during thermal conversion of cattle slurry digestates based only on existing literature. Further work is needed to better understand how key operating parameters impact the volatility of macronutrient elements and the volatility of heavy metals, that may contaminate the ash (or char) residues produced in thermal processes and therefore make challenging their utilisation. Furthermore, there is a clear need to study the chemical speciation of the macronutrients in the formed ash fractions to design a process that could be used to efficiently recycle nutrients from anaerobic digestates. Therefore, the primary objective of this study was to assess the influence of operating conditions, particularly gas atmosphere and temperature, on the volatility of macronutrient elements (P, N, K, Ca, Mg, and Na) and heavy metals (Zn, Cu, and Mn) during thermal conversion of cattle slurry-derived anaerobic digestates. A second objective was to identify the chemical forms of macronutrient elements (P, and K) that are retained in the formed combustion ashes. Finally, the implications of the results for utilising ash and char residues for soil improvement are discussed.

## Methods

### Anaerobic digestates

Two samples of anaerobic digestate were used in this work. Both samples were produced at a farm-scale biogas plant, located at the Maaninka Research station (63° 8’34.05"N, 27°19’7.23"E), about 40 km away from the city of Kuopio in the Northern Savonia region of Finland. The biogas plant uses cattle excrement, including faeces and urine, as feedstock and is operated by the Natural Resources Institute Finland. One sample, referred to as “AD-2018”, was produced during the summer of 2018. The other sample, referred to as “AD-2019”, was produced the following year, during the summer of 2019. Both samples were provided in 1 L air-tight plastic containers in the form of slurries. The moisture contents of the as-received slurries, determined by oven-drying 50 g test portions for 24 h at 105 °C, were both within the range 94–95% w/w.

Water was removed from the slurries in two stages. In the first stage, the slurries were centrifuged for 5 min at 5000 rpm. Solids were separated from the supernatant by decanting. This first stage of water removal simulates mechanical dewatering processes in which water-soluble inorganic elements are partly removed from the solids. Centrifugation reduced the moisture contents of the slurries to values within the range 88–89% w/w. The remaining moisture was completely evaporated from the dewatered slurries by oven-drying (48 h, 105 °C). Inorganic elements contained within the remaining moisture precipitate onto the digestates during oven-drying. The dried digestates were homogenised by milling with a laboratory knife mill equipped with a 1 mm screen. The homogenised samples of digestate were used for the release experiments and composition analyses.

### Release experiments

Nutrient element and heavy metal release experiments were carried out by heating small samples of the anaerobic digestates (0.5–0.7 g) in a laboratory-scale, fixed-bed tube reactor. The reactor setup features: a sample insertion probe for fast heating and quenching of samples in controlled gas atmospheres; a sample holder with porous walls to promote contact between biomass particles and gaseous reactants; a product gas dilution system for safe venting of flammable gas mixtures containing hydrogen; and a Fourier-transform infrared gas analyser (FTIR) for monitoring the concentrations of evolved product gases. Schematic diagrams and a detailed description of the reactor setup have been reported previously (Lane et al. 2020c). Reaction progress was monitored in select tests by measuring the concentrations of gaseous compounds, including CO_2_, CO, CH_4_, C_2_H_6_, C_3_H_8_, NO, N_2_O, HCN, NH_3_, SO_2_, and H_2_O, in the product gases and by analysing the ash (or char) residues for total carbon.

Samples were heated in a range of environments relevant to pyrolysis, combustion, and gasification processes (see Table S1 in the supplementary material for the complete experimental matrix). Combustion tests were carried out at two reaction temperatures, 800 °C and 1000 °C, in an atmosphere consisting of 1.5% O_2_ and 98.5% N_2_. The investigated combustion temperatures span (approximately) the range of operating temperatures used in commercial fluidised-bed biomass combustion and gasification technologies. Operating temperatures that are significantly lower than 800 °C, typically result in unacceptably low reaction rates. On the other hand, operating temperatures that are significantly higher than 1000 °C, typically result in problematic levels of ash melting. A low concentration of oxygen was used for the combustion experiments to prevent temperature overshoot caused by ignition of evolved volatiles during the devolatilisation phase of combustion. Samples were heated for total durations of 60 and 25 min at combustion temperatures of 800 °C and 1000 °C, respectively. These heating times were sufficiently long for complete (> 99.5%) burnout of carbon in the samples.

Pyrolysis and gasification tests were conducted at a consistent temperature (1000 °C) to enable the assessment of the impact of gas atmosphere on macronutrient and heavy metal volatility. The use of a high reaction temperature promotes complete destruction of pathogenic organisms in sludges and, in the case of pyrolysis processes, promotes formation of chars with beneficial properties for soil amelioration (Nicholas et al. 2023; Tomczyk et al. 2020). In the pyrolysis tests, samples were heated in a pure N_2_ atmosphere for a total duration of 20 min. Gasification tests were carried out in two gas atmospheres, pure CO_2_ and 10% H_2_ in N_2_. Samples were heated for a total duration of 120 min in all gasification tests. At the end of each reaction, the quenched ash (or char) residues were removed from the reactor, weighed to the nearest 0.01 mg, crushed to a fine powder with an agate mortar and pestle, and then analysed to determine the concentrations of macronutrient elements (P, K, Na, Mg, Ca, and N) and heavy metals (Zn, Cu, and Mn) in the residues. The release of these elements from the anaerobic digestates to the gas phase was then calculated by mass balance according to Eq. 1;

1 $$\:{R}_{i}\left(\%\right)=\left[1-\left(\frac{{W}_{r}}{{W}_{AD}}\right)\left(\frac{{C}_{r,i}}{{C}_{AD,i}}\right)\right]\times\:100$$

where R_*i*_ is the release of element *i* in weight%, W_*r*_ and W_*AD*_ are the weights (mg) of the residue and anaerobic digestate feedstock respectively, and C_*r, i*_ and C_*AD, i*_ are the concentrations (mg kg^− 1^, dry basis) of element *i* in the residue and anaerobic digestate, respectively.

### Composition analyses

A range of analytical techniques was used to measure element concentrations in the anaerobic digestates and in the thermal conversion residues, i.e., chars and ashes. Detailed descriptions of the techniques and evaluations of their accuracies when applied to various ashes have been reported previously (Lane et al. 2020a, c). The accuracies of the techniques when applied to biomass were verified by analysing a biomass standard reference material BCR 129 (see Table S2) and performing replicate analyses on the anaerobic digestates. The concentrations of C, N, H, and S were determined by oxidation of small samples (1.5 mg) in a micro elemental analyser. Water-soluble chlorine was determined by ion chromatography following leaching of chloride from samples (100 mg) with hot water. The concentrations of 24 metals and metalloids (Al, As, Bi, Ca, Cd, Co, Cr, Cu, Fe, K, Mg, Mn, Mo, Na, Ni, P, Pb, Sb, Si, Sn, Ti, Tl, V, and Zn) were determined using solution-based inductively coupled plasma mass-spectrometry following hot, pressurised digestion of samples (50–100 mg) in concentrated acids (HNO_3_, HF, and H_3_BO_3_). Method blanks and certified reference materials, BCR 129 (hay powder), BCR 176R (municipal solid waste incineration fly ash), and NIST 1648a (urban dust), were interspersed with analysis batches for quality control. The standard ash contents of the anaerobic digestates were determined by measuring sample weight loss following two consecutive stages of combustion in air (250 °C for 1 h and then 550 °C for 2 h) in a muffle furnace. The used method is a modified version of international standard ISO 18122:2015. The modified version uses smaller test portions (0.3 g) than specified in the standard method. Combustion ashes prepared at 800 °C and 1000 °C from AD-2018 were analysed for crystalline phases using powder X-ray diffraction (XRD). Details of the instrument setup, data acquisition parameters, analysis software, and diffraction databases have been reported previously (Lane et al. 2020c).

## Results and discussion

The chemical compositions of the two anaerobic digestates, AD-2018 and AD-2019 are presented in Table 1. Elemental composition and ash content variations between the two samples are minor. The main ash-forming elements in the samples are K, Si, Ca, P, Cl, Mg, S, Na, Fe, and Al (in descending order of concentration). The samples contain the following heavy metals in significant concentrations: Mn (299 and 333 mg kg^− 1^), Zn (223 and 298 mg kg^− 1^) and Cu (55 and 68 mg kg^− 1^). Most of the Cu and Zn in the digestates presumably originated from cattle feed, which contained around 15 mg kg^− 1^ of Cu and 76 mg kg^− 1^ of Zn (both values on a dry basis). These metals are added to livestock feeds to improve cattle health and immunity (Goselink and Jongbloed 2012). Other heavy metals (and metalloids), with the exception of Ti, are present in the digestates in relatively low concentrations (< 10 mg kg^− 1^). The volatilities of these elements were not considered in this study.

**Table 1:** Elemental compositions and ash contents of the anaerobic digestates

	AD-2018	AD-2019
C_a_ (% w/w)	41.9 ± 0.4	41.8 ± 0.4
H_a_ (% w/w)	5.26 ± 0.03	5.27 ± 0.03
N_a_ (% w/w)	2.14 ± 0.05	2.22 ± 0.05
S_a_ (% w/w)	0.44 ± 0.1	0.38 ± 0.08
Cl_b_ (% w/w)	1.11 ± 0.03	1.01 ± 0.03
K_c_ (% w/w)	2.9 ± 0.2	3.4 ± 0.2
Si_c_ (% w/w)	2.3 ± 0.3	1.4 ± 0.2
Ca_c_ (% w/w)	1.5 ± 0.1	1.7 ± 0.1
P_c_ (% w/w)	1.11 ± 0.05	1.20 ± 0.05
Mg_c_ (% w/w)	0.92 ± 0.04	1.03 ± 0.04
Na_c_ (% w/w)	0.49 ± 0.04	0.42 ± 0.04
Fe_c_ (% w/w)	0.201 ± 0.004	0.145 ± 0.003
Al_c_ (% w/w)	0.135 ± 0.005	0.087 ± 0.003
Mn_c_ (mg kg^− 1^)	299 ± 6	333 ± 7
Zn_c_ (mg kg^− 1^)	223 ± 33	298 ± 43
Ti_c_ (mg kg^− 1^)	107 ± 2	86 ± 2
Cu_c_ (mg kg^− 1^)	55 ± 3	68 ± 4
Ni_c_ (mg kg^− 1^)	10 ± 1	10 ± 1
Cr_c_ (mg kg^− 1^)	9.5 ± 1	6 ± 0.6
Mo^c^ (mg kg-1)	4.2 ± 1	3.5 ± 0.8
V_c_ (mg kg^− 1^)	3.2 ± 0.4	1.81 ± 0.2
Co_c_ (mg kg^− 1^)	2.2 ± 0.3	1.5 ± 0.2
Pb_c_ (mg kg^− 1^)	1.5 ± 0.2	1.1 ± 0.2
Sn_c_ (mg kg^− 1^)	1.6 ± 0.4	< 1
Bi_c_ (mg kg^− 1^)	< 1	1.5
Cd_c_ (mg kg^− 1^)	< 1	< 1
Sb_c_ (mg kg^− 1^)	< 1	< 1
As_c_ (mg kg^− 1^)	< 1	< 1
Tl_c_ (mg kg^− 1^)	< 1	< 1
ash_d_ (% w/w)	19.8 ± 0.2	20.0 ± 0.2

a = determined by combustion in a micro-elemental analyser

b = determined by IC following hot water extraction

c = determined by ICP-MS following pressurised acid digestion

d = ash content determined at 550 °C

**Table 2:** Char/ash yields following thermal conversion of AD-2018 at different operating conditions, and concentrations of macronutrient elements and heavy metals in the char and ash residues

Temperature	Reactant gas composition	Yield of ash or char	Concentration in char or ash residue									
(°C)		(%)	P (%)	K (%)	Na (%)	Ca (%)	Mg (%)	C (%)	N (%)	Zn (mg kg^− 1^)	Cu (mg kg^− 1^)	Mn (mg kg^− 1^)
800	1.5% O_2_ / bal. N_2_	16.9 ± 1.7	6.8 ± 0.3	11.7 ± 0.7	2.7 ± 0.1	9.2 ± 1.0	5.7 ± 0.4	0.13 ± 0.01	< 0.05	1260 ± 106	323 ± 25	1871 ± 129
1000	1.5% O_2_ / bal. N_2_	16.3 ± 1.6	6.8 ± 0.3	11.4 ± 0.7	2.6 ± 0.1	8.9 ± 1.0	5.7 ± 0.4	< 0.1	< 0.05	793 ± 67	321 ± 25	1889 ± 130
1000	N_2_	33.0 ± 3.3	3.1 ± 0.1	4.9 ± 0.3	0.6 ± 0.03	4.3 ± 0.5	2.6 ± 0.2	49.1 ± 4.8	1.02 ± 0.05	29 ± 2.4	162 ± 13	870 ± 60
1000	CO_2_	16.3 ± 1.6	7.6 ± 0.3	12.2 ± 0.8	2.8 ± 0.1	9.6 ± 1.0	6.0 ± 0.4	0.13 ± 0.01	< 0.05	776 ± 65	281 ± 22	1867 ± 129
1000	10% H_2_ / bal. N_2_	28.1 ± 2.8	1.7 ± 0.1	3.2 ± 0.2	0.2 ± 0.01	4.7 ± 0.5	2.8 ± 0.2	48.6 ± 4.7	0.38 ± 0.02	28 ± 2.4	155 ± 12	1026 ± 71

The yields of ash (or char) following thermal conversion of AD-2018 in different operating conditions are presented in Table 2. Also presented in Table 2 are the measured concentrations of macronutrient elements and heavy metals in the ashes and pyrolysis char. Ash/char yields and ash/char composition data for AD-2019 is presented in the supplementary material (see Table S3). Ash yields following combustion in 1.5% O_2_ / 98.5% N_2_ and CO_2_-gasification were between 15 and 20% lower than the standard ash contents. This result is attributed to the low combustion temperature (550 °C) used to determine the standard ash contents, which can result in reduced decomposition of carbonates (Mlonka-Mędrala et al. 2020) and reduced release of volatile inorganic species such as Cl (Johansen et al. 2011).

### Release of macronutrient elements

**Fig. 1:**
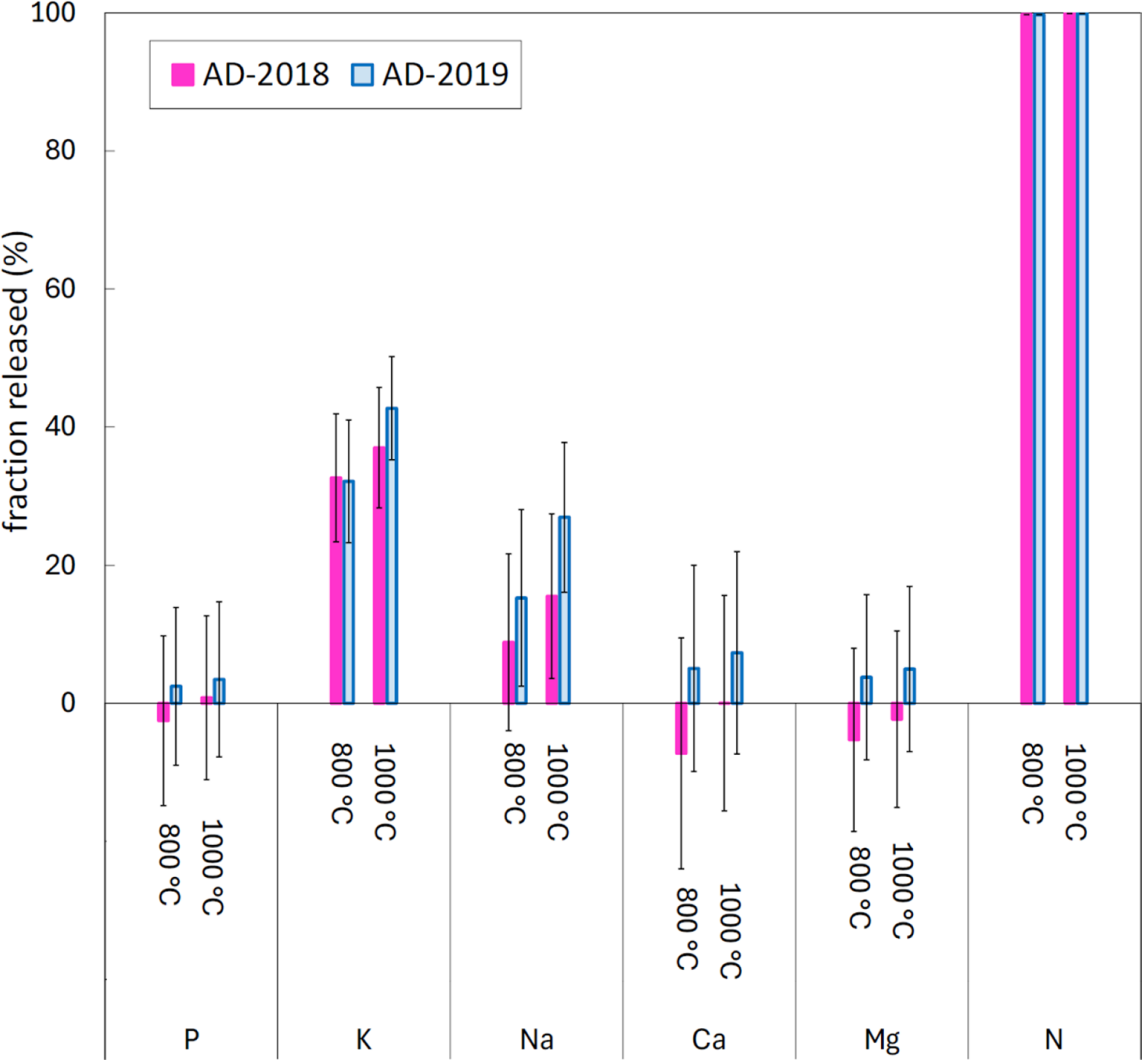
Fractional release of macronutrient elements to the gas phase during combustion of the anaerobic digestates at 800 °C and 1000 °C

**Fig. 2:**
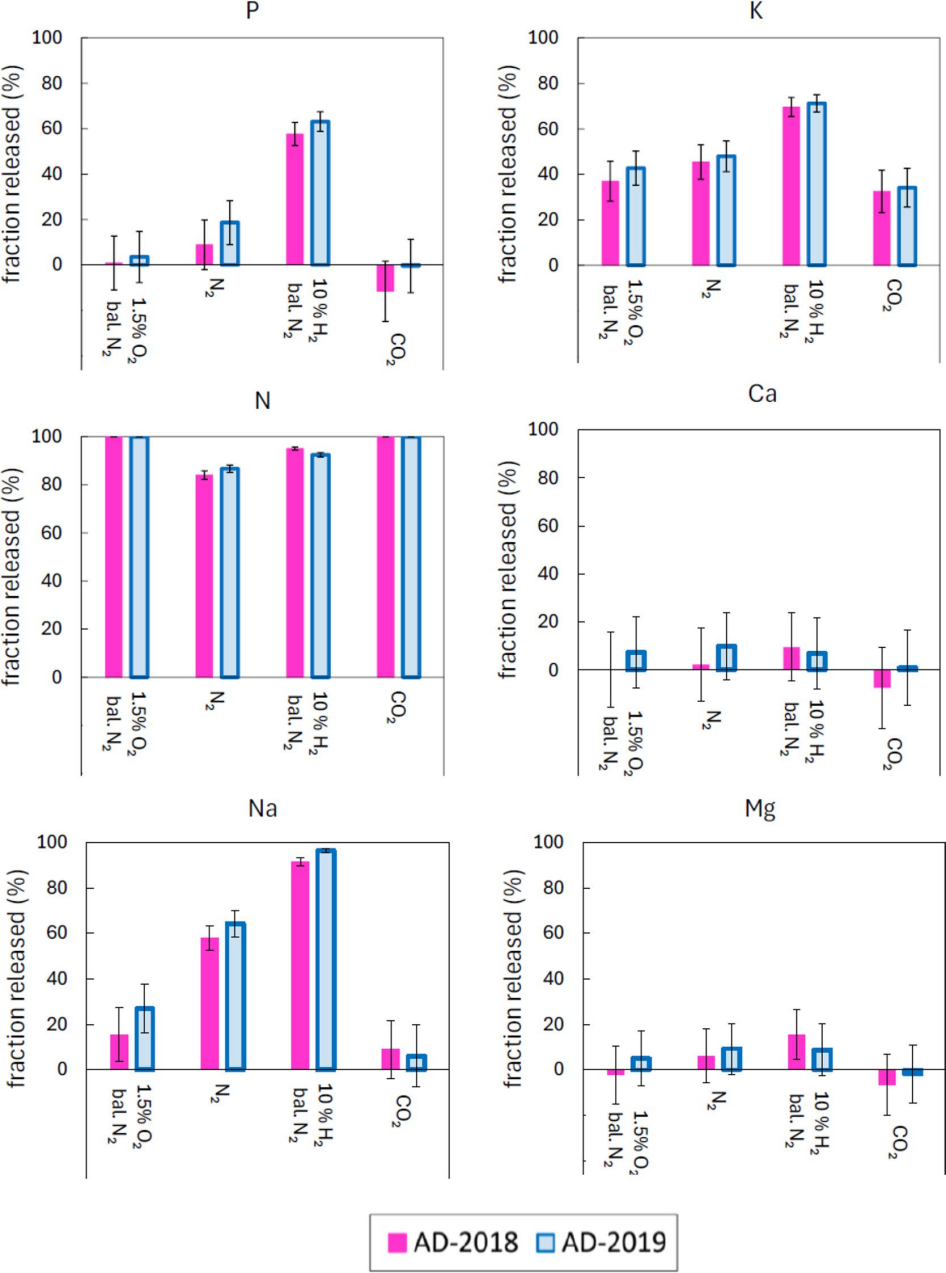
Fractional release of macronutrient elements to the gas phase during thermal conversion of anaerobic digestates at 1000 °C in different gas atmospheres

The fractional release of macronutrient elements to the gas phase during combustion in 1.5% O_2_ / 98.5% N_2_ is presented in Fig. 1. Results are presented for both samples of anaerobic digestate at two combustion temperatures, 800 °C and 1000 °C. Differences in release results between the two anaerobic digestate samples are minor. The impact of different gas atmospheres, pure N_2_, 1.5% O_2_ in N_2_, pure CO_2_, and 10% H_2_ in N_2_, on the release of these elements at 1000 °C is shown in Fig. 2. Low negative release values are shown for some tests. The low negative release values are attributed to analysis errors. The errors in the release values were evaluated by propagation of the errors in the elemental concentration measurements and ash (or char) yield measurements which were based on two standard deviations of the measured quantities. Errors were found to diminish with increasing release.

Phosphorous was completely retained in the ashes following combustion (both temperatures) and following CO_2_-gasification at 1000 °C. This agrees with previous studies that indicate only minor volatilization of P during combustion of sewage sludges (Falk et al. 2020; Häggström et al. 2023). In contrast, a moderate amount of P (~ 8– 20%) was released to the gas phase when the anaerobic digestates were pyrolyzed in pure N_2_ at 1000 °C. The fractional release of P was even greater (~ 58– 64%) following conversion of the anaerobic digestates in the reducing atmosphere consisting of 10% H_2_ in N_2_. This indicates that reducing conditions promote volatilisation of P from anaerobic digestates. The analysis of carbonaceous emissions during N_2_-pyrolysis of AD-2018 (see Figure S1) showed significant concentrations of CO and CH_4_ in the product gases. Peak concentrations of CO and CH_4_ during pyrolysis were ~ 3% v/v and ~ 0.4% v/v respectively. Significant concentrations of H_2_ (not measured) are also expected in the pyrolysis product gases. Thus, the release of P was clearly associated with reducing conditions during conversion of the anaerobic digestates.

The extent of release of the alkali metals, K and Na, varied greatly (32–71% for K and 6–96% for Na) over the range of investigated operating conditions. In combustion conditions, K was more volatile than Na. The release of K during combustion in 1.5% O_2_ / 98.5% N_2_ ranged from 32 to 33% at 800 °C and ranged from 37 to 43% at 1000 °C. The release of Na during combustion in the same atmosphere ranged from 9 to 15% at 800 °C and ranged from 16 to 27% at 1000 °C. The extent of release of K and Na was greater following pyrolysis in pure N_2_ and was greater again following thermal conversion in the reducing atmosphere consisting of 10% H_2_ in N_2_. The release of K and Na at 1000 °C was lowest when the anaerobic digestates were heated in CO_2_. The impact of the different gas atmospheres on volatility was greater for Na than for K. In general, the release of K and Na during thermal conversion of biomass is largely influenced by the formation of alkali metal containing silicates and phosphates that retain the alkali metals in coarse ash fraction (Sippula et al. 2017; Hedayati et al. 2021).

Calcium and magnesium had either low positive or low negative release values in all investigated operating conditions. This indicates that Ca and Mg are either not released at all or are released in only minor quantities. The low negative values are a result of experimental uncertainties.

Nitrogen was completely released (> 99.5%) to the gas phase during thermal conversion of the anaerobic digestates in the two atmospheres which contain oxygen (1.5% O_2_ in N_2_ and pure CO_2_). On the other hand, thermal conversion of the anaerobic digestates at 1000 °C in the pure N_2_ atmosphere and in the atmosphere consisting of 10% H_2_ in N_2_ resulted in incomplete release of N (84–87% release in pure N_2_ and 93–95% release in 10% H_2_ / 90% N_2_). Measurements of nitrogenous compounds in the emissions of anaerobic digestate combustion (see Figure S2) showed high concentrations of NO and N_2_O. Emissions of oxides of nitrogen (NO_x_) in practical biomass-fired systems are caused mainly by oxidation of biomass-derived nitrogen rather than reactions involving atmospheric N_2_ (Glarborg et al. 2003). One or more control measure, e.g., air staging and selective catalytic reduction (SCR) of NO_x_, will most likely be needed to reduce emissions of NO_x_ to acceptable levels during the conversion of anaerobic digestates derived from cattle slurries in industrial-scale installations.

**Fig. 3:**
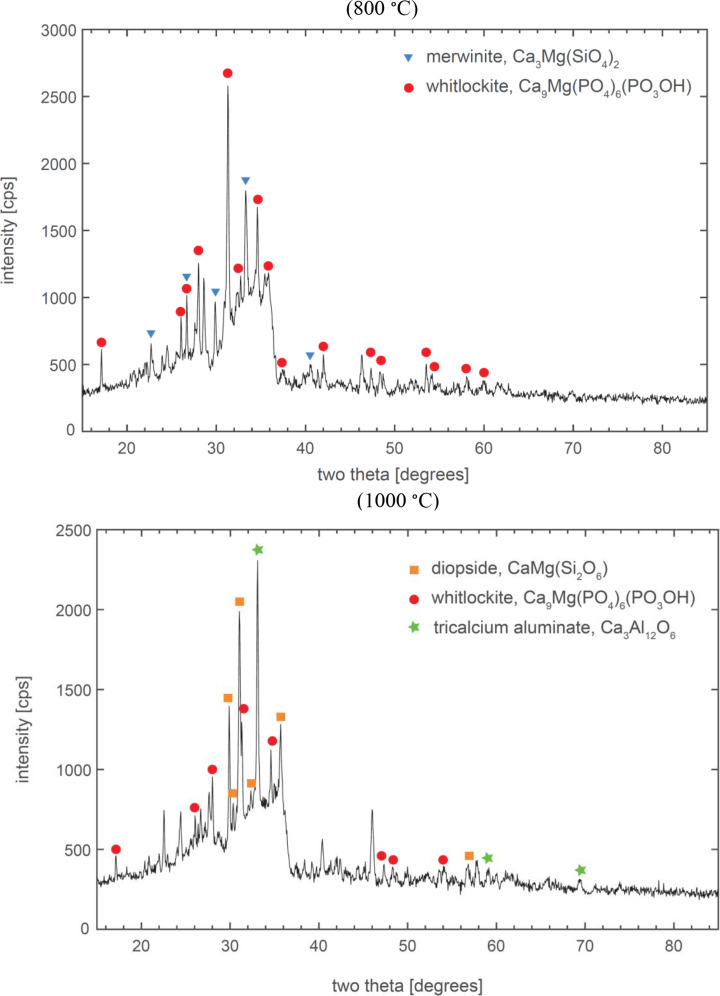
XRD spectra for combustion ashes prepared at 800 °C (top panel) and 1000 °C (bottom panel)

The XRD spectra for the combustion ashes prepared from AD-2018 are presented in Fig. 3. Two major crystalline phases were identified in the combustion ash prepared at 800 °C: merwinite (Ca_3_Mg(SiO_4_)_2_) and whitlockite (Ca_9_(K, Mg, Fe)(PO_4_)_6_(PO_3_OH). Three major crystalline phases were identified in the combustion ash prepared at 1000 °C: diopside (CaMgSi_2_O_6_), tricalcium aluminate (Ca_3_Al_12_O_6_), and whitlockite (Ca_9_(K, Mg, Fe)(PO_4_)_6_(PO_3_OH). Possible minor phases in the combustion ash prepared at 800 °C (not shown in Fig. 3) include: potassium magnesium phosphate (KMgPO_4_), polyhalite (K_2_Ca_2_Mg(SO_4_)_4_·2H_2_O), and antigorite (Mg_3_(Si_2_O_5_)(OH)_4_). Polyhalite was also identified as a possible minor phase in the combustion ash prepared at 1000 °C. The XRD analyses show that a substantial portion of P in the anaerobic digestates forms whitlockite during combustion at both 800 °C and 1000 °C. A large range of phosphates, which contain varying proportions of Ca, Mg, and K, have been reported by previous investigators in combustion ashes derived from other types of P-rich biomass, such as sewage sludge, meat bone meal, and cereal grains (Li et al. 2013; Lindström et al. 2007; Öhman et al. 2003; Skoglund et al. 2014). The formation of phosphates with high molar ratios of alkaline earth metals to K, such as whitlockite, can be considered advantageous with respect to the operation of industrial thermal processes, since these phosphates generally melt at higher temperatures than K-rich phosphates and consequently, tend to alleviate rather than exacerbate ash-related operational issues such as fouling, deposition, and, in the case of fluidised-bed reactor technologies, bed agglomeration (Lindström et al. 2007; Skoglund et al. 2014; Steenari et al. 2009). The presence of alkali metals in a whitlockite mineral structure has also been suggested to be positive regarding the plant bioavailability of phosphorus in ashes (Herzel et al. 2020; Falk et al. 2020).

### Release of heavy metals

**Fig. 4:**
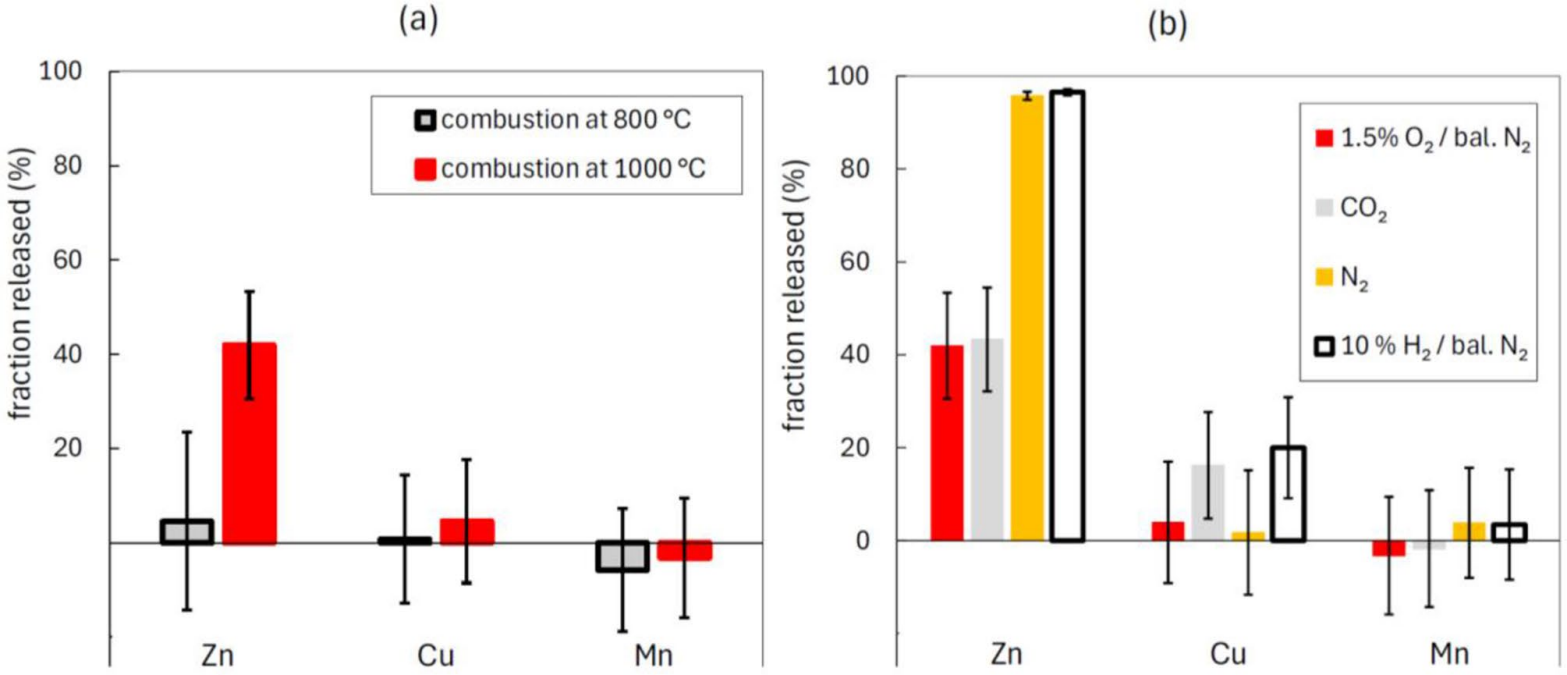
Fractional release of heavy metals to the gas phase during thermal conversion of AD-2018. Subplot (**a**) shows the impact of combustion temperature and subplot (**b**) shows the impact of gas atmosphere at 1000 °C

The fractional release of Zn, Cu, and Mn to the gas phase during thermal conversion of AD-2018 is presented in Fig. 4 for different operating conditions. The volatility of Zn varied greatly over the range of investigated operating conditions and was largely impacted by both temperature and gas atmosphere. Zinc had low volatility during combustion at 800 °C but was partly released to the gas phase during combustion and CO_2_-gasification at 1000 °C (~ 40–45%). The release of Zn was greatest (~ 96–97%) when the anaerobic digestate was heated in the reducing gas (10% H_2_ / 90% N_2_) and inert gas (pure N_2_) atmospheres. The high release of Zn during pyrolysis in pure N_2_ is attributed to local reducing conditions caused by formation of CO from the decomposition of carbon. The increased volatility of zinc in reducing conditions is consistent with thermodynamic equilibrium analyses of zinc speciation during combustion of biomass in fuel-rich and fuel-lean conditions (Elled et al. 2008; Sørum et al. 2003). Reducing gases, including CO and H_2_, reduce solid compounds of zinc to atomic zinc gas whereas oxidising atmospheres promote formation of non-volatile silicates and aluminates of Zn (Lane et al. 2020b; Sørum et al. 2003; Sinclair 2005).

The release of Mn was at most minor (< 20%) in all investigated operating conditions. Small fractions of Cu were released during CO_2_-gasification (~ 16% released) and during thermal conversion in the atmosphere consisting of 10% H_2_ in N_2_ (~ 20% released). The release of Cu was at most minor (< 20%) in the pyrolysis and combustion atmospheres (pure N_2_ and 1.5% O_2_ in N_2_).

### Implications for application of cattle slurry-derived ash and char residues onto soils

The inorganic constituents of biomass are separated into different ash (or char) fractions in industrial-scale thermal processes. Ashes that are separated from the product gases downstream of the reacting bed of biomass are generally enriched in volatile elements. These ashes are collectively referred to as fly ashes. Ash fractions that are collected below the reacting bed of biomass are enriched in non-volatile elements. These ashes are generally referred to as bottom ashes, or bed ashes in the case of fluidised-bed reactor technologies.

The partitioning of P in cattle slurry-derived digestates between bottom ashes and fly ashes will likely depend largely on the composition of the gas phase in contact with the reacting bed of anaerobic digestate. Given that the volatility of P in oxidising and inert gas atmospheres is low, it is expected that P will largely report to bottom ashes in commercial combustion processes. Previous investigators (e.g., Skoglund et al. 2014) have shown P to report mainly to coarse ash fractions during the combustion of biomass derived from municipal sewage sludges in laboratory-scale fluidised-bed reactors. However, the presence of local reducing zones in combustion processes, e.g., at the entrance of grate-fired combustors, may cause at least partial volatilisation of P and reduced recovery of P in bottom ashes. Nordin et al. (2020) compared the extent of transfer of P to bottom ashes during the combustion of municipal sewage sludge in two reactor types: an 8 MW_th_ grate-fired boiler and a fixed-bed, laboratory-scale furnace with excess supply of oxygen to the sample. The authors reported lower transfer of P to bottom ashes in the grate-fired boiler where reducing zones are encountered. In gasification processes, which typically have high concentrations of gas phase reductants, it is expected that P will volatilise to a significant extent and condense on fly ash fractions.

The laboratory-prepared combustion ashes were approximately 6-fold more concentrated in P than the dried anaerobic digestates and contained around 7% P w/w. Similar concentrations of P could be expected in the bottom ashes produced in industrial-scale combustors. Phosphorous concentrations in the laboratory-prepared combustion ashes are comparable with concentrations of P in low-grade phosphate rock ore. The global average grade of phosphate rock ore has been estimated to be around 10% P w/w, or 22.5% P_2_O_5_ (Van Kauwenbergh 2010), however, economic grades can be as low as 2.5% P w/w. According to the XRD analyses, a major part of phosphorus was present in the combustion ashes as mixed cation whitlockite minerals. These minerals generally have low phosphorus solubilities. Nevertheless, they have been reported to have suitable properties as slow-release fertilisers (Kumpiene et al. 2016; Mackay et al. 2017). Recent studies show that co-combustion or co-gasification of sludges with alkali metal-rich biomass causes an increase in the alkali metal content of formed whitlockite minerals (Herzel et al. 2020; Nordin et al. 2020). It is understood that this increases the bioavailability of P in ashes (Stemann et al. 2015; Herzel et al. 2020). Furthermore, the addition of dry biomass to moist anaerobic digestates increases the heating value of the combustion feedstock. Therefore, co-combustion of mixtures of moist anaerobic digestates with dry, alkali metal-rich biomass could potentially be used as a strategy to improve combustion performance and the fertiliser properties of the bottom ashes. Another potential strategy to increase the bioavailability of P in ashes is to treat the ashes in a thermochemical process with addition of a sodium salt. This strategy has been shown to convert whitlockite minerals to a mineral (buchwaldite) that makes P more bioavailable (Stemann et al. 2015).

Potassium is partially released to the gas phase in thermal processes and is expected to report to both bottom ash and fly ash in significant quantities. Higher temperatures and reducing gas atmospheres favour the release of K to the gas phase and are expected to increase the yield of K in fly ashes.

Zinc and copper are both essential micronutrients for plants (Alloway 2013). The addition of controlled quantities of these elements to soils can potentially be beneficial if the soil is deficient in these elements. However, high concentrations of Zn and Cu can have toxic effects on plants and on soil dwelling organisms (Alloway 2013; Nagajyoti et al. 2010; Reichman 2002). For this reason, the upper limits of the concentrations of Cu and Zn in biomass ashes for application onto agricultural and forest soils are limited by EU-level and national regulations (see Table 3 for limit values). Bottom ashes produced from combustion of cattle slurry-derived anaerobic digestates are expected to contain acceptable levels of Cu and Zn for application on agricultural and forest soils in Finland.

**Table 3:** Upper limits of Cu and Zn in biomass ash for application on agricultural and forest soils in European countries. Limit values are compared with global averages in topsoil and concentrations measured in the anaerobic digestate derived combustion ashes

	Cu (mg kg^− 1^, dry basis)	Zn (mg kg^− 1^, dry basis)
Global average in topsoil (2013)	14	62
**Anaerobic digestate derived combustion ashes**		
Combustion at 800 ºC	323 ± 25	1260 ± 106
Combustion at 1000 ºC	321 ± 25	793 ± 67
**Upper limit for ash application on agricultural soils**		
EU regulation 2019/1009	600	1,500
Sweden	600	800
Finland	600	1,500
**Upper limit for ash application on forest soils**		
Finland	700	4,500
Sweden	400	7,000

Fly ashes produced from thermal conversion of anaerobic digestates derived from cattle slurry have potential to contain high concentrations of the macronutrients, P and K, provided that the anaerobic digestates are exposed to reducing gaseous environments during thermal conversion. Such environments were found to promote significant volatilisation of these two elements. While fly ashes have potential to contain high concentrations of both P and K they will almost certainly be contaminated with high levels of Cl. It is expected that fly ashes will contain prohibitive levels of Cl given the high concentrations of Cl in the anaerobic digestates (1.0 and 1.1% w/w) and the high volatility of Cl at temperatures encountered in thermal processes (Björkman and Strömberg 1997; Johansen et al. 2011; Lane et al. 2015a). High concentrations of Cl in fly ashes are expected to prevent direct application of the fly ashes to soils due to the risk of increasing soil salinity.

Nitrogen was substantially released from the anaerobic digestates during both combustion and gasification. The nitrogen released in these processes forms gaseous species that are expensive to capture in industrial-scale installations, including N_2_, NO, N_2_O, NH_3_, and HCN (Glarborg et al. 2003; Leppälahti and Koljonen 1995). Given the high contents of N in the anaerobic digestates (2.1 and 2.2% w/w) it is recommended that practical installations be designed and operated in a way that promotes high conversion of fuel-N to N_2_ and minimal conversion of fuel-N to deleterious nitrogenous products, particularly NO and N_2_O, e.g. by employing air staging, selective noncatalytic reduction, or selective catalytic reduction.

A small fraction of N (~ 13–16%) was retained in the solid char residues following pyrolysis of the anaerobic digestates at 1000 °C. While the application of biomass derived chars to soils can significantly reduce emissions of NO and N_2_O from soils, this is not universally true for all biochar-soil combinations (Van Zwieten et al. 2009; Zheng et al. 2012). Additional research is needed to assess the potential of pyrolysis to mitigate emissions of NO and N_2_O.

## Conclusions

Phosphorous had low volatility in all atmospheres except the reducing atmosphere consisting of 10% H_2_ in N_2_. Phosphorous was retained in the combustion ashes as mixed cation whitlockite minerals. The volatilities of K, Na, and Zn were significantly impacted by temperature and were lowest in the oxygen-containing atmospheres (1.5% O_2_ in N_2_ and pure CO_2_). Copper, manganese, calcium, and magnesium had low volatilities in all investigated operating conditions. Bottom ashes produced from combustion of cattle slurry digestates are expected to meet legislative requirements for application onto agricultural and forest soils in Finland. Co-combustion of cattle slurry digestates with dry, alkali metal-rich agricultural residues is a potential strategy to improve both combustion performance and the bioavailability of phosphorous in the ashes. Further research is recommended to assess the benefits and limitations of this strategy.

## Electronic supplementary material

Below is the link to the electronic supplementary material.

Supplementary Material 1

Supplementary Material 2

## Data Availability

The datasets used and/or analysed during the current study are available from the corresponding author on reasonable request.
