# Supplementary material for: Fates of nutrient elements and heavy metals during thermal conversion of cattle slurry-derived anaerobic digestates
Source: Bioresour Bioprocess. 2024 Dec 30;11(1):115. doi: 10.1186/s40643-024-00828-7 (PMC11683038; doi:10.1186/s40643-024-00828-7)
Supplement: Supplementary file 1 — Supplementary Material 1 [file 40643_2024_828_MOESM1_ESM.docx]

# **Fates of nutrient elements and heavy metals during thermal conversion of cattle slurry-derived anaerobic digestates**

Daniel J. Lane, Olli Sippula, Jorma Jokiniemi, Mikko Heimonen, Niko M. Kinnunen, Perttu Virkajärvi, Narasinha Shurpali

**Supplementary Material**

**Table S1.** Experimental matrix

| test ID | sample ID | temperature | reactant gas composition | reaction time | ash/char analyses | | | | product gas analysis |
| --- | --- | --- | --- | --- | --- | --- | --- | --- | --- |
|  |  | (°C) |  | (min) | macronutrient elements | heavy metals | total carbon | powder XRD |  |
| 1 | AD-2018 | 800 | 1.5% O_2_ / bal. N_2_ | 25 | X | X | X | X |  |
| 2 | AD-2018 | 1000 | 1.5% O_2_ / bal. N_2_ | 60 | X | X | X | X | X |
| 3 | AD-2018 | 1000 | N_2_ | 20 | X | X | X |  | X |
| 4 | AD-2018 | 1000 | 10% H_2_ / bal. N_2_ | 120 | X | X | X |  |  |
| 5 | AD-2018 | 1000 | CO_2_ | 120 | X | X | X |  |  |
| 6 | AD-2019 | 800 | 1.5% O_2_ / bal. N_2_ | 25 | X |  | X |  |  |
| 7 | AD-2019 | 1000 | 1.5% O_2_ / bal. N_2_ | 60 | X |  | X |  |  |
| 8 | AD-2019 | 1000 | N_2_ | 20 | X |  | X |  |  |
| 9 | AD-2019 | 1000 | 10% H_2_ / bal. N_2_ | 120 | X |  | X |  |  |
| 10 | AD-2019 | 1000 | CO_2_ | 120 | X |  | X |  |  |

**Table S2.** Analysis of inorganic macronutrient elements and heavy metals (Zn, Cu and Mn) in the standard reference material BCR 129 (hay powder).

|  |  | **Concentration** | **Recovery** |
| --- | --- | --- | --- |
|  | **measured isotope** | **(mg/kg)** | **(%)** |
| **K** | 39 | 31122 | 92 |
| **Ca** | 42 | 5533 | 86 |
| **P** | 31 | 2407 | 102 |
| **Mg** | 24 | 1372 | 95 |
| **Na** | 23 | 3443 | 99 |
| **Fe** | 57 | 113 | 99 |
| **Al** | 27 | 101 | 90 |
| **Mn** | 55 | 79 | 110 |
| **Zn** | 68 | 37 | 115 |
| **Cu** | 65 | 9 | 89 |

**Table S3.** Char/ash yields following thermal conversion of AD-2019 at different operating conditions, and concentrations of macronutrient elements in the char and ash residues.

| temperature | reactant gas composition | yield of ash or char | concentration in char or ash residue (wt. %) | | | | | | |
| --- | --- | --- | --- | --- | --- | --- | --- | --- | --- |
| (°C) |  | (%) | P | K | Na | Ca | Mg | C | N |
| 800 | 1.5% O_2_ / bal. N_2_ | 16.7 ± 1.7 | 7.0 ± 0.3 | 13.8 ± 0.7 | 2.2 ± 0.1 | 9.6 ± 1.0 | 5.9 ± 0.4 | 0.80 ± 0.01 | <0.1 |
| 1000 | 1.5% O_2_ / bal. N_2_ | 16.4 ± 1.6 | 7.0 ± 0.3 | 11.8 ± 0.6 | 1.9 ± 0.1 | 9.6 ± 1.0 | 6.0 ± 0.4 | <0.1 | <0.1 |
| 1000 | N_2_ | 32.9 ± 3.3 | 3.0 ± 0.1 | 5.3 ± 0.3 | 0.50 ± 0.02 | 4.6 ± 0.5 | 2.8 ± 0.2 | 47.0 ± 4.9 | 0.90 ± 0.04 |
| 1000 | CO_2_ | 16.4 ± 1.6 | 7.3 ± 0.3 | 13.5 ± 0.6 | 2.4 ± 0.1 | 10.2 ± 1.1 | 6.4 ± 0.4 | 0.25 ± 0.02 | <0.1 |
| 1000 | 10% H_2_ / bal. N_2_ | 29.2 ± 2.9 | 1.5 ± 0.1 | 3.3 ± 0.2 | 0.10 ± 0.01 | 5.4 ± 0.6 | 3.2 ± 0.2 | 54.9 ± 5.3 | 0.60 ± 0.03 |

|  |
| --- |
|  |
|  |
|  |
|  |

**Figure S1.** Carbonaceous emissions from pyrolysis (N_2_) and combustion (1.5% O_2_ / bal. N_2_) of AD-2018. Initial sample masses were 0.53 and 0.71 g for pyrolysis and combustion respectively.

|  |
| --- |
|  |
|  |
|  |
| **Figure S2.** Nitrogeneous emissions from pyrolysis (N_2_) and combustion (1.5% O_2_ / bal. N_2_) of AD-2018. Initial sample masses were 0.53 and 0.71 g for pyrolysis and combustion respectively. |
