# Supplementary material for: Fates of nutrient elements and heavy metals during thermal conversion of cattle slurry-derived anaerobic digestates
Source: Bioresour Bioprocess. 2024 Dec 30;11(1):115. doi: 10.1186/s40643-024-00828-7 (PMC11683038; doi:10.1186/s40643-024-00828-7)
Supplement: Supplementary file 2 — Supplementary Material 2 [file 40643_2024_828_MOESM2_ESM.pdf]

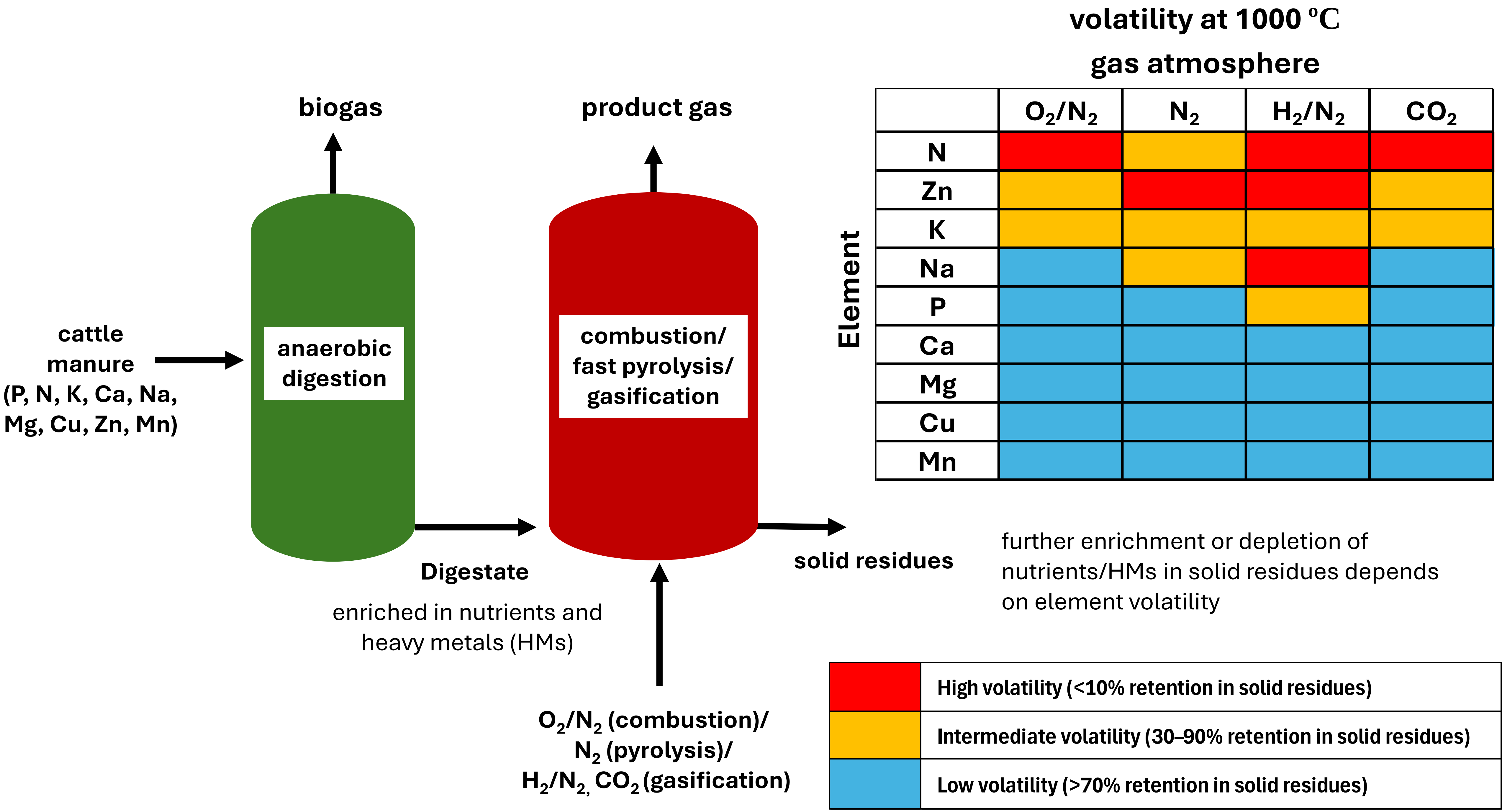

volatility at 1000 °C
gas atmosphere
biogas
product gas
O_2/N_2
N_2
H_2/N_2
CO_2
N
Zn
K
Na
P
Ca
Mg
Cu
Mn
Element
cattle manure (P, N, K, Ca, Na, Mg, Cu, Zn, Mn)
anaerobic digestion
combustion/ fast pyrolysis/ gasification
Digestate
enriched in nutrients and heavy metals (HMs)
solid residues
further enrichment or depletion of nutrients/HMs in solid residues depends on element volatility
O_2/N_2 (combustion)/ N_2 (pyrolysis)/ H_2/N_2, CO_2 (gasification)
High volatility (<10% retention in solid residues)
Intermediate volatility (30–90% retention in solid residues)
Low volatility (>70% retention in solid residues)
